# Genetic Characterization of *Plasmodium* Putative Pantothenate Kinase Genes Reveals Their Essential Role in Malaria Parasite Transmission to the Mosquito

**DOI:** 10.1038/srep33518

**Published:** 2016-09-20

**Authors:** Robert J. Hart, Emmanuel Cornillot, Amanah Abraham, Emily Molina, Catherine S. Nation, Choukri Ben Mamoun, Ahmed S. I. Aly

**Affiliations:** 1 Tulane University, Department of Tropical Medicine, New Orleans, LA 70112, USA; 2 Institut de Biologie Computationnelle, Université Montpellier, 34095 Montpellier, France; 3 Yale University School of Medicine, Section of Infectious Diseases, New Haven, CT 06520, USA

## Abstract

The metabolic machinery for the biosynthesis of Coenzyme A (CoA) from exogenous pantothenic acid (Vitamin B_5_) has long been considered as an excellent target for the development of selective antimicrobials. Earlier studies in the human malaria parasite *Plasmodium falciparum* have shown that pantothenate analogs interfere with pantothenate phosphorylation and block asexual blood stage development. Although two eukaryotic-type putative pantothenate kinase genes (*PanK1* and *PanK2*) have been identified in all malaria parasite species, their role in the development of *Plasmodium* life cycle stages remains unknown. Here we report on the genetic characterization of *PanK1* and *PanK2* in *P. yoelii*. We show that *P. yoelii* parasites lacking either *PanK1* or *PanK2* undergo normal asexual stages development and sexual stages differentiation, however they are severely deficient in ookinete, oocyst and sporozoite formation inside the mosquito vector. Quantitative transcriptional analyses in wild-type and knockout parasites demonstrate an important role for these genes in the regulation of expression of other CoA biosynthesis genes. Together, our data provide the first genetic evidence for the importance of the early steps of pantothenate utilization in the regulation of CoA biosynthesis and malaria parasite transmission to *Anopheles* mosquitoes.

Despite major efforts to control malaria, the disease continues to have significant health and economic impacts worldwide with millions of humans infected annually, and more than 500,000 deaths mostly caused by *Plasmodium falciparum*^1^,^2^. The widespread distribution of parasite clones that are resistant to a large panel of antimalarials has limited the classes of chemicals which can be further developed to combat drug resistance^3^. Therefore, studies aimed to identify new vulnerabilities in the parasite metabolic functions and to discover novel classes of inhibitors that can specifically and selectively target such metabolic functions and block parasite development at different stages of its life cycle are greatly needed.

The water-soluble Vitamin B_5_ (pantothenic acid) plays a critical role in cellular metabolism of living organisms by serving as the main precursor for the synthesis of coenzyme A (CoA) (Fig. 1A). CoA is an essential cofactor for a large number of metabolic processes including fatty acid biosynthesis, cellular oxidation, and carbohydrate and amino acid metabolism^4^. Studies in *P. falciparum* have shown that the parasite cannot synthesize pantothenic acid *de novo* and relies on the uptake of exogenous pantothenate for survival^4^,^5^,^6^,^7^,^8^,^9^. Once transported inside the parasite, pantothenic acid is rapidly phosphorylated to form phosphopantothenate (PP) by parasite encoded pantothenate kinases^10^. This reaction has been shown to be the rate-limiting step in CoA biosynthesis in many organisms^11^,^12^,^13^,^14^. Phosphopantothenate is subsequently converted into CoA by four enzymes PPCS (Phosphopantothenylcysteine Synthase), PPCDC (Phosphopantothenylcysteine Decarboxylase), PPAT (Phosphopantetheine Adenylyltransferase) and DPCK (Dephospho-CoA Kinase)^4^ (Fig. 1A).

Earlier metabolic analyses^5^,^6^,^15^ and more recent studies in *P. falciparum* have indicated that pantothenic acid uptake and utilization are absolutely crucial for asexual blood stage development of the parasite within human erythrocytes^7^,^8^,^9^,^10^,^16^,^17^. Consistent with these data, pantothenate analogs inhibit intraerythrocytic development of *P. falciparum*^16^,^18^,^19^. The potency of some of these compounds is reduced following supplementation with either exogenous pantothenic acid or CoA indicating their direct effect on CoA biosynthesis^9^,^20^. Moreover, it was shown that the enzyme pantetheinase, a vanin family protein present in serum and RBCs that typically hydrolyzes pantetheine into pantothenate in a reversible reaction, has been implicated in the degradation of promising pantothenate analogues^18^. Recently, a vanin-resistant analog has been reported and shown to inhibit the growth of *P. falciparum* 3D7 clone with an IC_50_ of ~20 nM, which is comparable to chloroquine, highlighting the promise of this pathway for development of new potent antimalarials^19^.

Other malaria parasites have been shown to use alternative routes for the synthesis or acquisition of CoA. Earlier studies in *P. lophurae* have shown that this avian parasite can scavenge host CoA for survival in ducks erythrocytes^6^,^21^. Similarly, genetic studies in the murine malaria parasite *P. yoelii* have shown that parasites lacking a candidate pantothenic acid transporter undergo normal asexual development in mouse erythrocytes, suggesting the presence of an alternative route for CoA biosynthesis in this parasite or an alternative pantothenate secondary transporter^22^.

Here we report the genetic characterization of two putative pantothenate kinase genes, in *P. yoelii*. We show that transgenic parasites lacking *PyPanK1* or *PyPanK2* undergo normal asexual development and sexual differentiation in mouse erythrocytes. However, these knockout parasites are severely deficient in ookinete development and completely blocked in their ability to produce sporozoites in *Anopheles* mosquitoes.

## Results

### Characterization and sequence analysis of *Plasmodium* putative pantothenate kinase genes *PanK1* and *PanK2*

**Figure 1:**
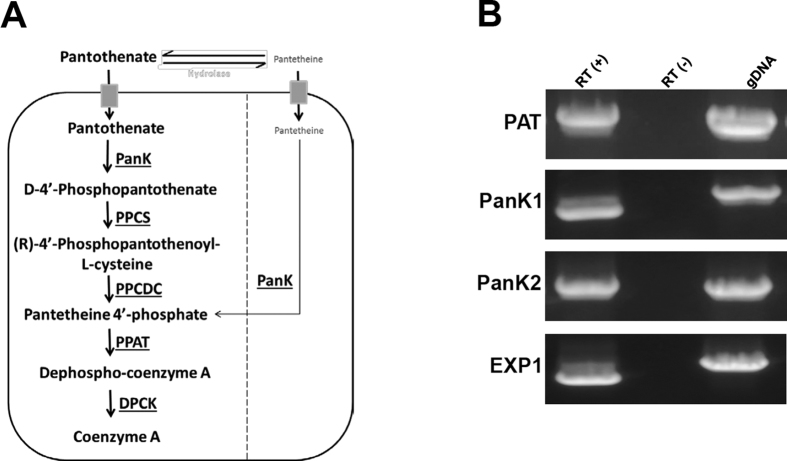
Model of the CoA biosynthesis machinery in the malaria parasite and transcription of *PanK1* & *PanK2* during blood stage development in *P. yoelii*. (**A**) Pantothenic acid is first transported into the parasite by a pantothenate transporter and then phosphorylated to form 4′-phosphopantothenate. Addition of cysteine to 4′-phosphopantothenate by a Phosphopantothenylcysteine Synthase (PPCS) produces 4′-phospho-N-pantothenolycysteine (PPC). Decarboxylation of PPC by a PPC decarboxylase (PPCDC) produces 4′-phosphopantetheine (PP). Adenylation of PP by Phosphopantetheine Adenylyltransferase (PPAT) produces Dephospho-CoA. The latter is phosphorylated by a Dephospho-CoA Kinase (DPCK) to produce CoA. (**B**) Evidence for *PyPanK1* and *PyPanK2* transcription during *P. yoelii* asexual blood stage development in mouse erythrocytes. RT-PCR analysis on total RNA isolated from wild-type infected blood (parasitemia ~1%) was performed using the primer pairs listed in Supplementary Table 1. Expression of the *P. yoelii PyEXP1* (Exported Protein1) and *PyPAT* known to be expressed in asexually replicating parasites was also determined as expression controls. Control PCR reactions on RNA in the absence of reverse transcription (−) or on genomic DNA were also performed.

The availability of genome sequence data from different species of malaria parasites made it possible to use sequences of bacterial, fungal, plant and mammalian CoA biosynthesis enzymes to search for their counterparts in the *Plasmodium* genome databases. All eukaryotic-type homologues of the enzymes of CoA biosynthesis have been identified in all *Plasmodium* species sequenced so far (http://mpmp.huji.ac.il/maps/coasynthesis.html). One *Plasmodium* homologue was identified for PPCS, PPCDC, PPAT and DPCK but two homologues were identified for Pantothenate kinase (PanK) (Fig. 1A). To date, biochemical characterization of the malarial pantothenate kinase activity has been determined only from parasite extracts. Although this activity has been thoroughly characterized in *P. falciparum*, its encoding genes have not yet been characterized. In order to determine whether *PanK1* and *PanK2* are expressed during *P. yoelii* asexual development in mouse erythrocytes, RT-PCR analyses were performed on total RNA isolated from erythrocytes infected with the wild-type (WT) 17XNL strain of *P. yoelii*. As shown in Fig. 1B, *PyPanK1* and *PyPanK2* cDNAs were amplified only in the presence of the reverse transcriptase but not in the absence of this enzyme. As a control, cDNAs of the *PyEXP1 (Exported Protein 1*) and *PyPAT* genes known to be expressed during blood stage development were also amplified (Fig. 1B).

**Figure 2:**
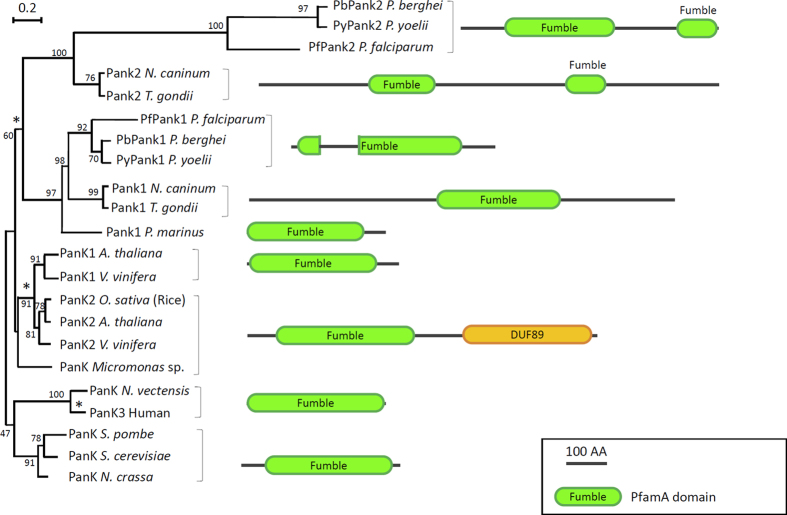
Sequence structure and evolution of Apicomplexan PanK1 and PanK2 proteins compared to confirmed pantothenate kinases. Schematic diagram showing eukaryotic pantothenate kinase protein organization from plants, protists and algae that are closely related to human PanK3. Apicomplexan PanKs are shown with *Plasmodium* conserved PanK1 and PanK2 that resemble PanK1 and PanK2 from *Neospora caninum* and *Toxoplasma gondii,* respectively. The phylogenetic analysis has been inferred by Maximum Likelihood, using Muscle and PhyML through *phylogeny.fr* “one click” option web interface. Scale bar indicates the number of substitution per site. Protein structures have been deduced from PfamA database and results from Pfam search engine. Star (*) indicate node where duplication of the PanK gene may have occurred during evolution of eukaryotes. The bootstrap values are shown next to the cluster of the tree.

**Figure 3:**
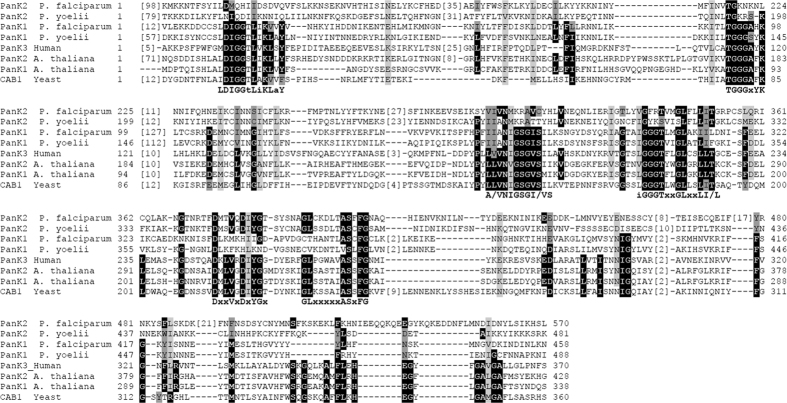
Conserved sequence signatures in *Plasmodium* and other known and putative eukaryotic pantothenate kinases. Pantothenate kinase fumble domain sequence organization. Residues that were described to be conserved between plant and human enzymes are shaded in black. Similar residues found at these positions are shaded in dark grey (hydrophobic: I, L, M, V; small: A, C, G, S, T; Negative: D, E; Positive: K, H, R; aromatic: F, W, Y). Other conserved residues according to Muscle multiple alignment are highlighted in light grey. Four highly conserved PanK motifs TGGGxYKF, A/VNIGSGI/VS, iGGGTxxGLxxLI/L and LDIGGS/TLiKLaY that are present in human and plant PanKs are also present in *Plasmodium* PanK1^24^, while *Plasmodium* PanK2 harbors the highly conserved PanK motifs DxxVxDxYGx and GLxxxxxASxFG.

Homologs of *Plasmodium* PanK proteins were found to be highly conserved in other apicomplexan parasites including *Toxoplasma gondii* and *Neospora caninum* (Fig. 2). Sequence analysis revealed that like their homologs in other eukaryotes, *Plasmodium* PanK1 and PanK2 proteins contain either one (PanK1) or two copies (PanK2) of the fumble (fbl) domain initially identified in the *Drosophila* fbl protein and shown to play an essential role in cell division^23^. The importance of this domain in the activity of pantothenate kinases has not yet been elucidated. Indeed, *Plasmodium PanK1* harbors all the signatures of active eukaryotic pantothenate kinases and contains four highly conserved motifs TGGGxYKF, A/VNIGSGI/VS, iGGGTxxGLxxLI/L and LDIGGS/TLiKLaY that are present in human and plant PanKs; the latter domain is critical for ATP binding and catalysis^24^ (Fig. 3). *Plasmodium* PanK2 proteins, however, lack these motifs but share instead two domains DxxVxDxYGx and GLxxxxxASxFG with human and plant PanKs (Fig. 3).

### PanK1 and PanK2 are not essential during *Plasmodium yoelii* intraerythrocytic development

**Figure 4:**
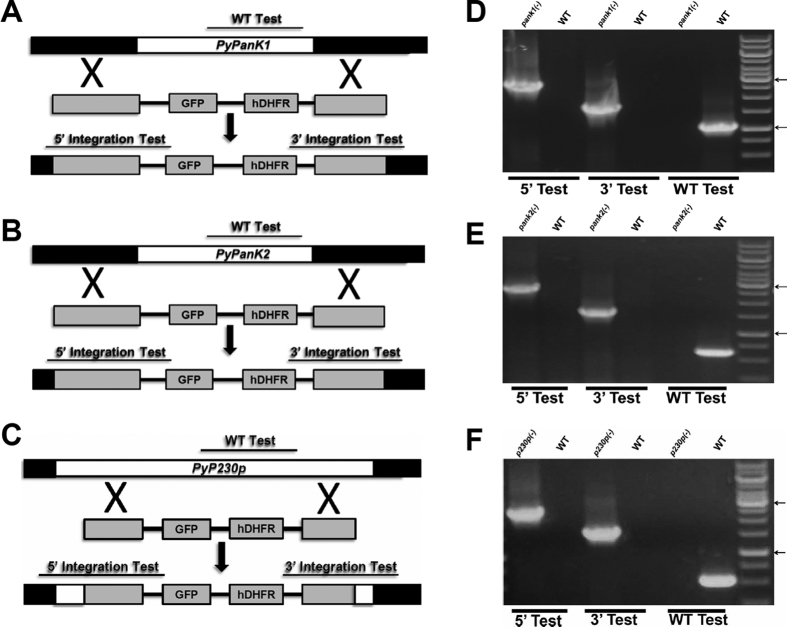
Targeted deletion of *PyPanK1* and *PyPanK2* genes. Schematic representation of the replacement strategy to generate *Pypank1*(−) parasites in (**A**), *Pypank2*(−) parasites in (**B**) and *Pyp230p*(−) parasites in (**C**). The endogenous *Py*PanK1, *Py*PanK2 and *Py*P230p genomic loci are targeted with replacement fragments containing the 5′ and 3′ *Py*PanK1 and *Py*PanK2 UTRs and *Py*P230p ORFs sequences, respectively, flanking the human DHFR positive selection marker and eGFP cassettes. The three constructs were transfected during the same experiment, where P230p serves as a transfection experiment and transfection vector control. Diagnostic WT-specific or integration-specific test amplicons are indicated by lines. 36-cycles PCR genotyping confirms the integration of gene-replacement construct using oligonucleotide primer combinations that can only amplify from the recombinant loci (5′ Test and 3′ Test). The WT-specific PCR reaction (WT) confirms the absence of WT parasites in *Pypank1*(−) in (**D**), in *Pypank2*(−) parasites in (**E**) and in *Pyp230p*(−) parasites in (**F**).The arrows show the size of DNA ladder bands of 3000 and 1000 bps, respectively.

**Figure 5:**
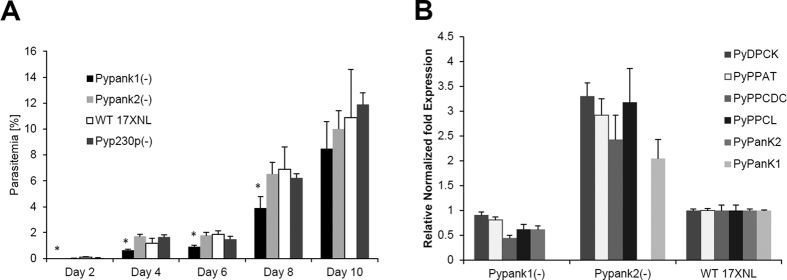
Non-essential roles and regulation of gene expression of CoA biosynthesis genes of *PanK1* and *PanK2* during *P. yoelii* blood stage development. (**A**) Average blood stage parasitemia (% infected erythrocytes out of >5000 cells counted) in groups of four BALB/c mice per strain following IV injection of 5000 infected erythrocytes confirms that deletion of *PyPanK1* or *PyPanK2* does not lead to any significant reduction in blood stage parasitemia compared to WT and WT-like *Pyp230p*(−) parasites only at day 10 pi. However, the deletion of *PyPanK1* slightly, but significantly, affected blood stage parasitemia at days 2, 4, 6 and 8 pi. (**B**) Deletion of *PyPanK2* results in upregulation of *PyPanK1* and other CoA biosynthesis genes transcripts, while deletion of *PyPanK1* results in a slight downregulation of CoA biosynthesis genes transcripts. Transcript levels of *PyPanK1* and *PyPanK2* as well as other genes involved in CoA biosynthesis (*PAT*, *PPCS*, *PPCDC*, *PPAT* and *DPCK*) in wild-type, *Pypank1*(−) or *Pypank2*(−) strains were estimated by real time-PCR from total RNA isolated from similar amounts of infected mouse blood with 1% parasitemia. Relative gene expression levels were normalized to *18S* ribosomal RNA expression in *P. yoelii* blood stages, and are presented as the mean of two independent experiments, error bars represent standard deviation.

Having demonstrated transcription of *PyPanK1* and *PyPanK2* during *P. yoelii* development in mouse erythrocytes, we then investigated whether their genetic disruption could alter parasite development or sexual differentiation in the mouse blood. Two targeting vectors harboring the 5′ and 3′ regions of *PyPanK1* and *PyPanK2* were thus constructed flanking the human DHFR positive selection marker for disruption of the genomic loci of *PyPanK1* and *PyPanK2*. The same targeting vector was used to disrupt the dispensable *PyP230p* locus as a control for the transfection vector and procedures. All vectors were linearized and transfected in the same transfection experiment with *PyPAT*, which was previously published^22^. Transgenic parasites were cloned and analyzed by PCR to confirm the genetic replacement events and the deletion of *PyPanK1* (Fig. 4D), *PyPanK2* (Fig. 4E) and *PyP230p* (Fig. 4F) genes. These parasites were then examined for their intraerythrocytic development in BALB/c inbred mice compared to WT following intravenous (IV) inoculation with 5 × 10^3^ iRBCs from each parasite genotype (Fig. 5A). Giemsa-stained thin blood smears were collected and parasitemia recorded on even days post infection (pi) until day 10, and then afterwards daily only to check for clearance, which occurred at days 17 or 18 pi. As proven from published reports^22^,^25^,^26^,^27^,^28^, transgenic parasites lacking the *PyP230p* gene exhibited normal development in mouse erythrocytes compared to WT parasites (Fig. 5A). However, there was a slight, but statistically significant, growth reduction of *Pypank1*(−) blood stage parasites compared to all other genotypes at days 2, 4, 6 and 8 pi, but not at day10 pi (Fig. 5A). This data shows that while the deletion of *PanK2* does not affect the growth of blood stage parasites, the deletion of *PanK1* has a very slight, but significant effect on the malaria parasite progression in mouse blood.

### Deletion of *PyPanK1* or *PyPanK2* results in major alteration of expression of CoA biosynthesis genes

To examine whether the viability of *PyPanK1* and *PyPanK2* single knockouts may be due to redundant function of pantothenate phosphorylation, we examined the expression of these genes as well as that of other CoA biosynthesis genes in each knockout by real time-PCR performed on RNA isolated from blood stage parasites collected from mice with similar parasitemia levels (1% parasitemia). As shown in Fig. 5B, deletion of *PyPanK2* resulted in a 2-fold increase in the expression of *PyPanK1* transcript levels, whereas deletion of *PyPanK1* resulted in a slight decrease in *PyPanK2* transcript levels (Fig. 5B). Interestingly, the transcript levels of PPCS, PPCDC, PPAT and DPCK were also increased by about 2-fold in *Pypank2*(−) and all of them decreased slightly in *Pypank1*(−) (Fig. 5B). This would indicate that PanK1 and PanK2 have a role in the regulation of gene expression of CoA biosynthesis enzymes at least at the transcriptional level, which could resemble the function of PanK as the rate limiting enzyme in other eukaryotes^11^,^12^,^13^,^14^. In order to confirm that these results were due to the absence of PanK genes and not due to subtle expression differences between transgenic parasites, we compared the transcription levels of *PanK1* and *PanK2* in addition to different other unrelated genes (*18S*, *EXP1* and *DHFR/TS*) in *Pyp230p*(−) parasites and WT parasites. RT-PCR analysis with similar amounts of RNA (60 ng) isolated from the same amount of blood extracted from similar infected blood parasitemia (~1%) confirmed that the transcription levels of *PanK1* and *PanK2* and other unrelated genes are similar in *Pyp230p*(−) parasites compared to WT parasites (Supplementary Figure 1). Collectively, these data indicate that the genes involved in CoA biosynthesis are co-regulated, at least at the transcriptional level, by a mechanism that is involving *PanK1* and *PanK2* expression.

### Parasites lacking *PyPanK1* or *PyPanK2* undergo normal sexual differentiation but cannot complete their development in the mosquito

**Figure 6:**
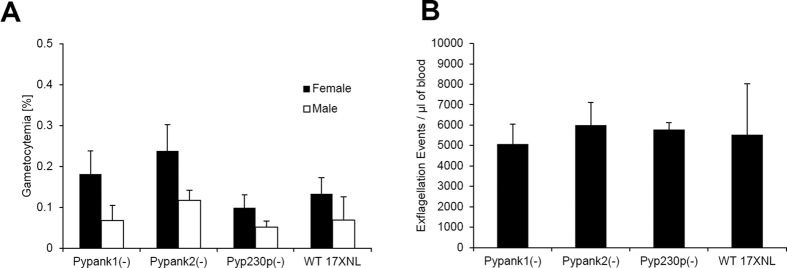
Parasites lacking *PyPanK1* or *PyPanK2* undergo normal sexual differentiation. (**A**) Average percentage of male and female mature gametocytes in groups of SW mice IV injected three days earlier with 10^6^ erythrocytes infected with either WT or knockout parasites (all are G5 passages). (**B**) Average number of male gamete exflagellation events per μl of mouse blood determined by a hemocytometer using 1:10 dilution of tail blood. The mean values for all parasite strains were analyzed with the One-Way Analysis-of-Variance (ANOVA) and statistical significance was set at a P value of <0.05. P values in experiments A and B were all >0.05. The results shown are the mean of three independent experiments with each independent experiment involving at least three mice per parasite strain, error bars represent standard deviation.

**Figure 7:**
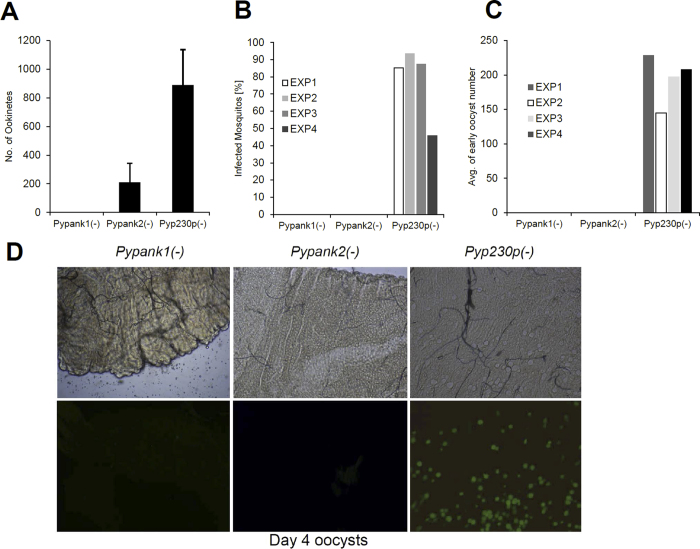
Ookinete and oocyst development are completely abolished in *Pypank1*(−) and *Pypank2*(−) parasites. (**A**) Average number of ookinetes identified in mosquitoes fed on blood infected with either wild type or knockout parasites 20 hours pmf. The P value of 0.0056 estimated in experiments C for the *Pypank2*(−) parasite was significant. The results shown are the mean of three independent experiments (**B**) Average percentage of mosquito midgut infection determined by the presence of early oocysts (day 4 pmf) for *Pypank1*(−), *Pypank2*(−) and *Pyp230p*(−) infected mosquitoes in four different independent experiments using fluorescence microscopy. Midguts with at least one oocyst were counted as infected. (**C**) Estimation of the Average number of early oocysts (day 4 pmf) from *Pypank1*(−), *Pypank2*(−) and *Pyp230p*(−) infected mosquitoes midguts in four different independent experiments using fluorescence microscopy. (**D**) Fluorescent microscopy images of midguts of *Anopheles* mosquitoes infected with *Pypank1*(−), *Pypank2*(−) and *Pyp230p*(−) (magnification 100X).

The viability of *Pypank1*(−) and *Pypank2*(−) parasites in mouse erythrocytes made it possible to assess the function of *Py*PanK1 and *Py*PanK2 in gametocyte development as well as in gametogenesis. Outbred SW mice were IV infected with 10^6^ *Pypank1*(−), *Pypank2*(−), *Pyp230p*(−) or *Py*WT infected red blood cells and blood samples were collected and analyzed at day 3 post-infection to determine the number of male and female gametocytes and male gametes. No significant differences in the number of male or female gametocytes (P ~ 0.66 and P ~ 0.35, respectively) or male gametes (P ~ 0.87) could be detected among all strains tested (Fig. 6A,B). To examine the importance of *Py*PanK1 and *Py*PanK2 during the development of ookinetes, female *Anopheles stephensi* mosquitoes were fed on infected mice displaying the highest male gamete exflagellation rate. After 20 hours post-mosquito feeding, mosquito midguts from at least 20 blood-fed female *Anopheles stephensi* were dissected and mature ookinetes were quantified (Fig. 7A). A dramatic reduction (P ~ 0.0056) in ookinete numbers was observed in knockout parasites lacking *Py*PanK1 or *Py*PanK2 compared to wild type parasites. Whereas an average 890 ookinetes were detected in control parasites, ~211 ookinetes were produced by *Pypank2*(−) and 0 by *Pypank1*(−) parasites (Fig. 7A). These data suggest that *Py*PanK1 plays an essential role in ookinete formation whereas the role of *Py*PanK2 in this phase of the parasite development is important but not essential.

In the mosquito midgut, *Plasmodium* ookinetes traverse the midgut epithelium before reaching the basal lamina and transforming into spherical oocysts 24–48 hours post-mosquito feeding^29^. Oocysts then undergo multiple mitotic divisions that result in the formation of hundreds of sporozoites from each oocyst. To investigate the importance of *Py*PanK1 and *Py*PanK2 in oocyst formation and sporozoite development, we took advantage of the fact that *Pypank1*(−)*, Pypank2*(−) and *Pyp230p*(−) parasites constitutively express eGFP. Midguts of mosquitoes fed on *Pypank1*(−)*, Pypank2*(−) and *Pyp230p*(−) parasites were dissected, and the number of oocysts were counted using fluorescence microscopy at day 4 post-mosquito feeding (Fig. 7B–D). Results from four independent experiments revealed that between 46 and 94% of dissected mosquitoes that fed on *Pyp230p*(−) control parasites were infected and contained between 145 and 228 oocysts (Fig. 7B,C). On the other hand, no oocysts could be detected out of 125 and 123 dissected mosquitoes fed on *Pypank1*(−) and *Pypank2*(−) infected blood, respectively (Fig. 7B–D). Interestingly, whereas the number of oocyst sporozoites in mosquitoes fed on mice infected with *Pyp230p*(−) parasites ranged between 11,388 and 24,000 average sporozoites per mosquito, only two abnormal sporozoite-like structures were detected in the midguts of *Pypank1*(−) infected mosquitoes and no sporozoites could be detected in mosquitoes fed on *Pypank2*(−) parasites by day 10 post-mosquito feeding (Table 1). Together, these data demonstrate a critical role for *PyPanK1* and *PyPanK2* during ookinete formation and ookinete-to-oocyst conversion.

## Discussion

Our studies provide the first genetic evidence that the two candidate pantothenate kinase genes of *P. yoelii* are critical for malaria transmission to the mosquito but that loss of one or the other does not alter asexual development or sexual differentiation. These findings are consistent with previous data that showed *P. yoelii* knockout parasites lacking the candidate pantothenate transporter *PyPAT* undergo normal asexual development in mouse blood^22^. The ability of different *Plasmodium* species to utilize different host precursors for the synthesis of CoA has been first reported several decades ago when it was shown that the development of *P. lophurae* in duck erythrocytes relies on the ability of this parasite to salvage CoA from its host^21^,^30^. However, the metabolically active and nucleated avian erythrocytes are different from mammalian anucleated erythrocytes, and thus it is less likely that CoA is salvaged by rodent malaria species from reticulocytes. This may suggest unknown alternative mechanisms for synthesis of CoA, possibly through pantothenate intermediates like pantetheine which can be reversibly synthesized from pantothenate through pantetheine hydrolases or panthetheinases^31^,^32^,^33^. Noteworthy, pantetheine is used as a primary substrate for CoA synthesis in some bacterial strains^4^. Moreover, CoA intermediates like phospho-pantetheine and dephospho-CoA have been shown to rescue the development of *P. lophurae* in a pantothenate or CoA free medium^34^. Together, these data suggest that an alternative mechanism for CoA biosynthesis is used by *P. yoelii* during its development in mouse blood.

While the exact alternative route for CoA synthesis in *P. yoelii* remains unknown, the finding that the initial steps for CoA synthesis are not essential in this parasite makes it an excellent model to study the role of the CoA biosynthesis genes in sexual differentiation or transmission to the mosquito. During *Plasmodium* transition from intracellular growth in the vertebrate blood to extracellular growth in the midgut of the mosquito vector, the parasite undergoes major physiological and morphological changes. After the mosquito takes up a blood meal that contains gametocytes, the male gametocyte undergoes up to three rounds of mitotic divisions and cytokinesis to produce up to eight highly motile gametes in the mosquito midgut in less than ten minutes^29^. Therefore, all nutritional needs for mitotic division, cytokinesis and fertilization must be present in female and male gametocytes before transmission to the mosquito, including the nutritional needs for CoA biosynthesis. Consistent with this model, male gamete exflagellation was similar between *Pypank1*(−), *Pypank2*(−) and WT parasites. On the other hand, the conversion of the spherical zygote into an elongated motile invasive ookinete is accomplished in ~24 hours while the parasite is not enclosed within a host cell. At this stage of the parasite development major differences could be noted between the two knockout strains. Analysis of the *Pypank1*(−) mutant strain demonstrated that *Py*PanK1 is essential for the formation of mature ookinetes as no ookinetes, and accordingly, no oocysts or sporozoites could be detected in the mosquito. The *Pypank2*(−) mutant, however, produced 20% less ookinetes than wild type parasites but failed to produce oocysts or sporozoites. These findings indicate that the expression of *Py*PanK1 in the *Pypank2*(−) mutant or *Py*PanK2 in the *Pypank1*(−) mutant is not sufficient for normal development of *P. yoelii* in the mosquito, and suggests that both proteins are required for optimal ookinete formation and oocyst development. Although it is unknown whether *Plasmodium* PanK1 and PanK2 enzymes form a complex required for activity, our expression analyses indicate that the transcription level of expression of *PyPanK1* and *PyPanK2* genes as well as that of other CoA genes is highly affected by the presence or absence of *PanK1* or *PanK2* transcripts. In particular, expression of all CoA biosynthesis genes was found to be up-regulated by 2 to 3 fold in the absence of *PyPanK2*. Whether this regulation is mediated by the effect of *PyPanK2* on the CoA biosynthetic pathway at the protein or metabolic levels remains to be further investigated. Due to the limited number of selectable markers available for knockout analyses of *P. yoelii in vivo*, transgenic parasites lacking both *PyPanK1* and *PyPanK2* genes were not produced. However, several attempts to knockout *PyPanK1* in the *Pypank2*(−) or *PyPanK2* in the *Pypank1*(−) genetic backgrounds did not result in viable parasites lacking both genes. These findings may indicate that such a double knockout is lethal in blood stage asexual development, or may also point out that the inability to obtain the double knockout was due to technical difficulty. Future knockouts and complementation analyses using recyclable markers and homologs from other *Plasmodium* species (for complementation analyses) may help better understand the function and regulation of this pathway in this parasite.

Although pantothenate phosphorylation activity can be measured from parasite extracts, so far all attempts to express individual recombinant active *Plasmodium* PanK1 and PanK2 have not been successful. Sequence analysis indicated that *Py*PanK2 lacks an ATP binding domain, and thus by itself may not catalyze a phosphorylation reaction^24^. We hypothesize that similar to other pseudokinases^35^, *Plasmodium* PanK2 may serve as a scaffold for a protein complex containing PanK1 and possibly other component of the CoA biosynthesis machinery to mediate metabolism of pantothenate and possibly other precursors used by this parasite for the synthesis of CoA. In the absence of an *in vitro* assay to determine the activity of recombinant *Plasmodium* PanK1 and PanK2, the *P. yoelii* knockouts generated in this study will help future metabolic and biochemical analyses to characterize the metabolism of CoA precursors in this parasite and help gain a better understanding of this process, and its inhibition by pantothenate analogs in human malaria parasites.

## Materials and Methods

### Experimental Animals, Parasites and Mosquitoes

Female Swiss Webster (SW) mice and BALB/c mice (6 to 8 weeks old) were purchased from Envigo Research Models and Services, formerly known as Harlan Laboratories, (Indianapolis, Indiana). All Animal experiments were conducted according to the approved protocols of the Institutional Animal Care and Use Committee (IACUC) of Tulane University. All other experimental protocols and the use of recombinant DNA were conducted according to the approved protocols of the Institutional Biosafety Committee (IBC) of Tulane University. Initial mosquito feeding experiments were conducted on *Pyp230p*(−) independent clones A4 and A5^22^, *Pypank1*(−) independent clones A2 and B2, and *Pypank2*(−) independent clones C4 and D4, and showed that only the A4 and A5 clones of *p230p*(−) produced oocyst sporozoites at day 10 post feeding, whereas all clones of *Pypank1*(−) and *Pypank2*(−) knockouts failed to produce oocyst sporozoites in two independent experiments. All subsequent detailed phenotypic analyses in mice and mosquitoes were performed, using *Pyp230p*(−) clone A5, *Pypank1*(−) clone A2 and *Pypank2*(−) clone D4 parasites, as previously described^22^,^36^.

### Generation of Transgenic Parasites

Targeted gene deletions induced by homologous recombination were performed as previously described^22^,^36^,^37^. Briefly, 5′ and 3′ UTR regions of the target genes were amplified from genomic DNA of *P. yoelii* 17XNL strain using primers described in Supplementary Table 1. The amplified fragments were inserted into the transfection plasmid pAA20 between *SacII* and *BamHI,* and *KpnI* and *HindIII*, respectively. The resulting targeting vectors were linearized with *SacII* and *KpnI* prior to transfection of *P. yoelii* 17XNL parasites by electroporation with Lonza Nucleofector II. Selection of resistant parasites and cloning of transfectants were performed as previously described^25^,^38^. To confirm the disruption of the targeted genes, gDNA was extracted from parasites clone and diagnostic PCR analyses were performed using primers pairs listed in Supplementary Table 1 and a 36 cycle PCR program (94° for 30 seconds, 55° for 30 seconds and 60° for 4 minutes).

### Transcriptional Analyses

Total RNA was extracted from 1.5 ml infected blood from 2 infected mice (parasitemia ~1% with >90% asexually replicating parasites). RNA was then purified, and treated with DNAse DNA-*free* kit (Ambion). Reverse transcription was performed using SuperScript III kit (Invitrogen) on 3μg of RNA. For analysis of expression of *PyPanK1* and *PyPanK2* during *P. yoelii* intraerythrocytic life cycle, 60 ng of RNA (1μl from RT(+) or RT(−) cDNA) was used in each RT-PCR reaction. As a control for primer amplification and for the splicing size difference, gDNA (~10 ng DNA as a template per reaction) was tested with each primer set used. The following primers sets were used in the RT-PCR analyses in Fig. 1B and in Supplementary Figure 1: *PAT* (39 and 40), *PanK1* (57 and 58), *PanK2* (67 and 68), *EXP1* (rtEXP1-F and rtEXP1-R), *DHFR/TS* (rtDHFR-F and rtDHFR-R) and *18S* (rt18S-F and rt18S-R). All RT-PCR reactions were conducted for 36 cycles (PCR cycle conditions: 94° for 30 seconds, 55° for 30 seconds and 60° for 2 minutes). For qPCR analyses to determine levels of expression of CoA biosynthesis genes the following primers were used: *18S* RNA (real18S-F and real18S-R), *PyPanK1* (realPanK1-F and realPanK1-R), *PyPanK2* (realPanK2-F and realPanK2-R), *PyPAT* (realPAT-F and realPAT-R), *PyPPCS* (realPPCS-F and realPPCS-R), *PyPPCDC* (realPPCDC-F and realPPCDC-R), *PyPPAT* (realPPAT-F and realPPAT-R), *PyDPCK* (realDPCK-F and realDPCK-R). Each qPCR reaction was performed in triplicates and repeated twice using iTaq Universal SYBR Green Supermix (Bio-Rad) according to the manufacturer’s instructions. Amplification followed by melt curve was performed with the C1000 Touch Thermal cycler with CFX96 Real-Time System (Bio-Rad). Relative transcript abundance was normalized to *18S* ribosomal RNA expression. Data was analyzed using Bio-Rad CFX Manager 3.1. All primer sequences are listed in the Supplementary Table 1.

### Protein sequence analysis

Protein sequences were obtained from Uniprot^39^ and used to search for homologs by BLAST search of Uniprot and NCBI databases^40^ and for domain identification in the Pfam database version 27^41^. The Pank protein Uniprot IDs used to draw the phylogenetic tree include Human (PANK3_HUMAN), *Nematostella vectensis* (A7SEX1_NEMVE), Yeasts (*Saccharomyces cerevisiae*: PANK_YEAST, *Schizosaccharomyces pombe*: PANK_SCHPO), Fungi (*Neurospora crassa*: Q7SEX7_NEUCR), *Arabidopsis thaliana* (Pank1: PANK1_ARATH, PanK2: PANK2_ARATH), *Vitis vinifera* (Pank1: D7TDH2_VITVI, Pank2: F6GSM7_VITVI), *Oryza sativa* (Pank2: B8BDU9_ORYSI), *Micromonas* sp. (C1FDA4_MICSR), *P. falciparum* (PanK1: Q8ILP4_PLAF7, PanK2: Q8IL92_PLAF7), *P. yoelii* (PyPanK1: V7PMS1_9APIC, PanK2: V7PFL4_9APIC), *P. berghei* (PbPanK1: Q4YYH4_PLABA, PanK2: Q4YWX0_PLABA), *Toxoplasma gondii* (TgPanK1: S8EU95_TOXGO, PanK2: S8EZV3_TOXGO), *Neospora caninum* (NcPanK1: F0VPI2_NEOCL, PanK2: F0VKG1_NEOCL), *Perkinsus marinus* (C5L4T0_PERM5). Phylogenetic analysis was performed using Phylogeny.fr “One click” option^42^. The pipeline include Gblock analysis^43^. The implemented PhyML software^44^ estimates maximum likelihood phylogeny. Default option include the use of the approximate Likelihood-Ratio Test (aLRT) with 100 bootstraps. Phylogenetic analysis was performed on a set of about 70 relevant positions depending on the set of proteins that were selected.

## Additional Information

**How to cite this article**: Hart, R. J. *et al.* Genetic Characterization of *Plasmodium* Putative Pantothenate Kinase Genes Reveals Their Essential Role in Malaria Parasite Transmission to the Mosquito. *Sci. Rep.* **6**, 33518; doi: 10.1038/srep33518 (2016).

## Supplementary Material

Supplementary Information

## Figures and Tables

**Table 1:** Oocyst Sporozoite formation is abolished in parasites lacking *PyPanK1* or *PyPanK2*.

	Pypank1(−)	Pypank2(−)	Pyp230p(−)
Average number of midgut oocyst sporozoites per mosquito (day 10 post mosquito feeding)			
Experiment 1	0	0	11,388
Experiment 2	0	0	15,680
Experiment 3	0	0	24,000
