# Supplementary material for: Genetic Characterization of Plasmodium Putative Pantothenate Kinase Genes Reveals Their Essential Role in Malaria Parasite Transmission to the Mosquito
Source: Sci Rep. 2016 Sep 20;6:33518. doi: 10.1038/srep33518 (PMC5028760; doi:10.1038/srep33518)
Supplement: Supplementary Information [file srep33518-s1.pdf]

Supplementary Online Information:

Genetic Characterization of *Plasmodium* Putative Pantothenate Kinase Genes Reveals Their Essential Role in Malaria Parasite Transmission to the Mosquito.

Robert J. Hart[1], Emmanuel Cornillot[2], Amanah Abraham[1], Emily Molina[1], Catherine S. Nation[1], Choukri Ben Mamoun[3] and Ahmed S. I. Aly*[1].

[1] Tulane University, Department of Tropical Medicine, New Orleans, LA 70112, USA.

[2] Institut de Biologie Computationnelle, Université Montpellier, 34095 Montpellier, France.

[3] Yale University School of Medicine, Section of Infectious Diseases, New Haven, CT 06520, USA.

*To whom correspondence should be addressed:

Ahmed S. I. Aly, Tulane University, Department of Tropical Medicine, New Orleans, USA.

Email: aaly@tulane.edu

Supplementary Table 1

Primer sequences (5’-3’)
39: AGTATGGCATTTCATAAATCTTTGGCTGTAGTA;
40: ACTATTGTTTCATTATTATTATCAATTATACTT;
41: GGCCGCGGATGATAAACCATATTTTGAAGAAATTATTAGTGA;
42: TCCGGATCCGCTTATTCTGGATTTTCAAATGAATAGTTAATAA;
43: GCCCAAGCTTCTTTAAGAAATTATGAACAATTCAAGCAATCCAA;
44: TCCGGTACCTTCCTAATATATATGATTTATAAATTAATTTGAT;
45: GAAGATTTTATCATTATTCGAGTCAGAGTACATCA;
46: ACACGAACCCAAAGAAATCGGCAAGCTAAAACTA;
47: ATTATATGGATGTGATTTTTCAGGTAATTCTAA;
48: CATACTCAAATATCTTTTTAGTAGTTCCTCAATA;
51: GGCCGCGGGAATAATAAAATCTTTTACACTATTATATCTGCT;
52:TCCGGATCCTTCCAATTAAATCGATATATTTTTACTAATCAT;
53:TGCCAAGCTTTTTTTAATATGTAAATAGTAAAATAAAGAATATATG;
54:TCCGGTACCGTTAATGACATTGGTACGTGTGTGTATACTATTTC;
55:ACATTATTAATAATAATAGGTCATAAAATATAAGG;
56:TCTAAACAAAATATCAACTTTTTTTGTTGTTTCCT;
57:GGCCGCGGAGCACAAATACCAATACAAATCAAATCACCACTT;
58:TCCGAATTCAGAAAAAAAAACCTCCGATAACTCCTAAAAATCC;
61: GGCCGCGGTGGGGCAAGACAAATATAAAACCTTTAAAACCCAT;
62: TCCGGATCCAAAGGAGCATTATACTCCTATGATATTCCCCAT;
63: TGCCAAGCTTTTTTTGAAACACTTTTTTTTTTTAAGTTATATTTG;
64: TCCGGTACCTTGACAACAAATATTATTTTTAATTTGAAAACG;
65: TGTAGTTAAGTGATCATATATAGTAACTGAGTT;
66: TGAAGCTCTATATATTTTGACTAAATACGAAATT;
67: GCAAAGTAAAGATGGTGAATTTTCATTAAACGAA;
68: TCAATAAAAAAAGGGATATAACTGATTTATACCC;
16: ATGTCCATTAACATCACCATCTAATTCAACAAG;
17: GTGTTCTTTCTGATGTTCAAGAAGAAAAAGGTA;
real18S-F: GCGGCGAGTACGCTATATCC;
real18S-R: CATGTCGAAGATGCGAGAAA;
realPanK1-F: GTAGATATGTCTTTATAAACTTGAGG;
realPanK1-R: AGAACTTCGGGGTATTGTATCATCT;
realPanK2-F: CTTTGATATGTCTGTTGGCGATATT;
realPanK2-R: ATTCATAGCTGACCCGAAACA;
realPAT-F: GTCCGCTTTAGGAGGAGTATTAG;
realPAT-R: ACGGTACTCTACCAAACATAGC;
realPPCS-F: TGGATAATATATTGGGCGAACT;
realPPCS-R: CCTCCAGAGGTGACCAAA;
realPPCDC-F: CGAGGTGATGATATCTTGCATATAGA;
realPPCDC-R: TCCAACAACGACAAATAGAGGT;
realPPAT-F: TGCCCTTGATATTCGAGATATGTT;
realPPAT-R: GTGTTCGATTTGTTCGCTCTTAAT;
realDPCK-F: CATCAGTTCGGTAGTGGTGAA;
realDPCK-R: ACCTACAGCTATACCTCCTGTAA;
rtEXP1-F: AACAAGTATGGTAAAAATGGCAAATATGGCTCC;
rtEXP1-R: GTTTTGAACATTTGGGGTGTTAGATGGTGCAGG;
rt18S-F: GCGGCTTAATTTGACTCAACACGGGGAAACTCA;
rt18S-R: CCGCAAAAGAGCAAAAGGTAAACCAATTGATTA;
rtDHFR-F: GTAAGAATATGGGAAGCTAATGGAACAAGAGAA;
rtDHFR-R: AGCATTACCCAATACATGTATAAATTCAGCTGG.

__Supplementary Figure 1__

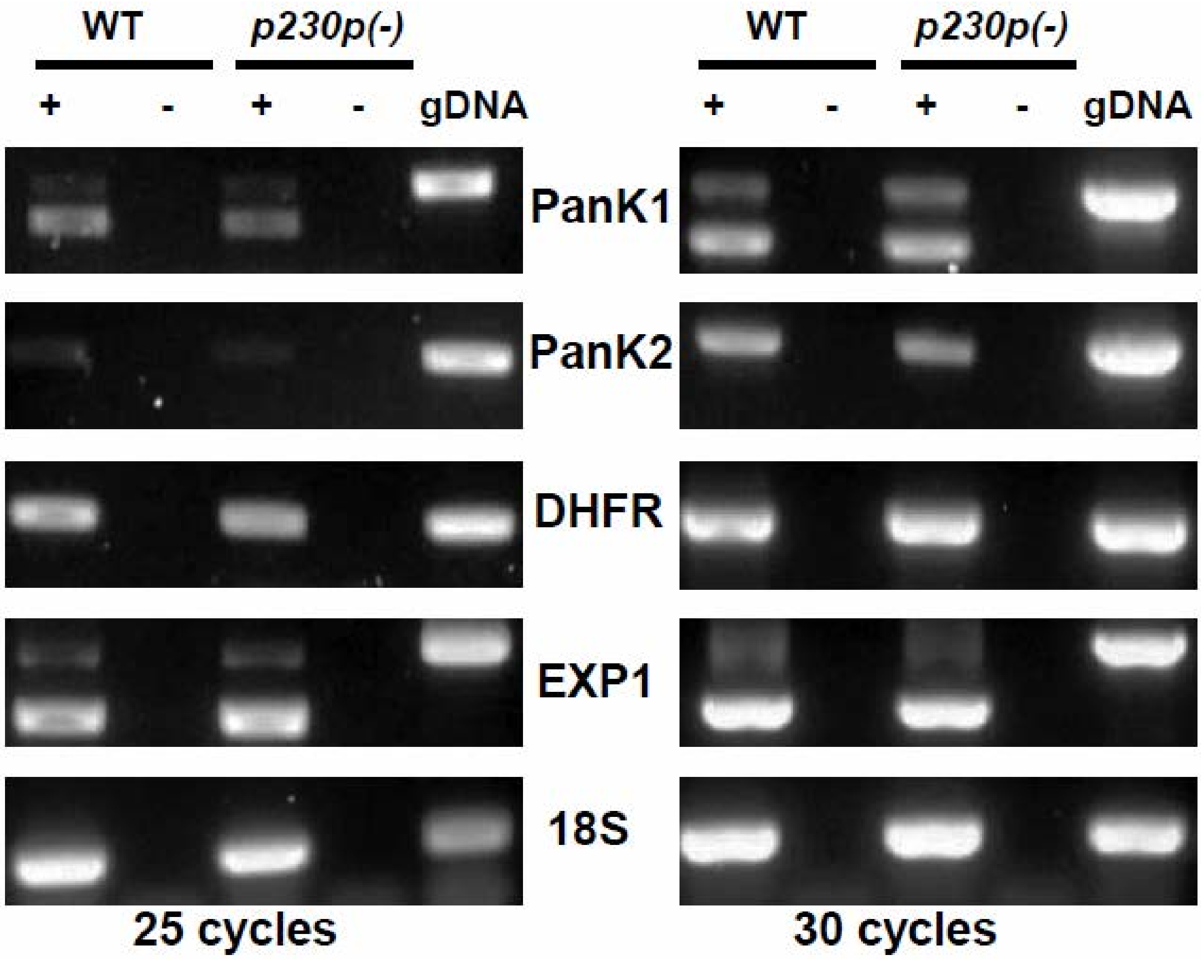

Supplement. Figure1: Similar transcription levels of *PanK1, PanK2* and different unrelated genes (*18S*, *EXP1* and *DHFR/TS*) during the blood stage cycle of *P. yoelii* 17XNL WT strain compared to *Pyp230p(-)* control WT-like strain. RT-PCR analysis of total RNA isolated from blood stage parasites of 17XNL WT and *Pyp230p(-)* strains (with similar infected blood parasitemia of ~1%) was performed using the primer pairs listed in Supplementary Table1. Control RT-PCR reactions on RNA in the absence of reverse transcription (-) and on genomic DNA were also performed. Similar amounts of RNA of 60 ng was used for each RT +/- reaction, while 20 ng of WT gDNA was used for each genomic PCR reaction.
